# Effectiveness of a new sensorized videolaryngoscope for retraining on neonatal intubation in simulation environment

**DOI:** 10.1186/s13052-020-0774-z

**Published:** 2020-02-03

**Authors:** Alice Covelli, Serena Bardelli, Rosa T. Scaramuzzo, Emilio Sigali, Massimiliano Ciantelli, Marta Del Pistoia, Alessia Longo, Selene Tognarelli, Arianna Menciassi, Armando Cuttano

**Affiliations:** 1 Centro di Formazione e Simulazione Neonatale “NINA”, U.O. Neonatologia, Dipartimento Materno-Infantile, Azienda Ospedaliero Universitaria Pisana, Pisa, Italy; 2 U.O. Neonatologia, Dipartimento Materno-Infantile, Azienda Ospedaliero Universitaria Pisana, Via Roma 67, Pisa, Italy; 3 The BioRobotics Institute, Scuola Superiore Sant’Anna, Pontedera, Italy

**Keywords:** Retraining, Neonatal intubation, Skill trainer, Simulation, Sensorized

## Abstract

**Background:**

In recent years, medical training has significantly increased the use of simulation for teaching and evaluation. The retraining of medical personnel in Italy is entrusted to the program of Continuous Education in Medicine, mainly based on theoretical training. The aim of this study is to assess whether the use of a new sensorized platform for the execution of the neonatal intubation procedure in simulation environment can complement theoretical retraining of experienced health professionals.

**Methods:**

Neonatal intubation tests were performed using a commercial manikin and a modified video-laryngoscope by the addition of force and position sensors, which provide the user with feedback when the threshold is exceeded. Two categories carried out the simulation tests: anesthesiologists and pediatricians. The categories were divided into three groups each, and various configurations were tested: the first group of both specialists carried out the tests without feedback (i.e. control groups, gr. A and A1), the second groups received sound and visual feedback from the instrument (gr. B and B1) and the third ones had also the support of a physician expert in the use of the instrument (gr. C and C1). The instrumentation used by pediatricians was provided in a playful form, including a game with increasing difficulty levels.

**Results:**

Both in the case with feedback only and in the case with humans support, anesthesiologists did not show a specific trend of improvement. Pediatricians, in comparison with anesthesiologists, showed a positive reaction to both the presence of feedback and that of experienced personnel. Comparing the performance of the two control groups, the two categories of experienced doctors perform similar forces. Pediatricians enjoyed the “Level Game”, through which they were able to test and confront themselves, trying to improve their own performance.

**Conclusions:**

Our instrument is more effective when is playful and competitive, introducing something more than just a sound feedback, and allowing training by increasing levels. It is more effective if the users can adapt their own technique to the instrument by themselves, without any external help.

## Background

For several years, the word “retraining” in America has been being related to a protocol of reintegration into clinical practice for doctors who have been out of work for some time [1]. The Office of Professional Medical Conduct requires the participation to a retraining program to renew the license of individuals clinically inactive for more than a decade [2]. According to a report published in 2008 by the American Medical Association Council on Medical Education, two or more years away from clinical practice are sufficient to require the physician to participate in a re-entry program, in which the essential clinical skills are evaluated to develop a specific plan of recovery [3].

In recent years, medical training has significantly increased the use of simulation as a didactic and evaluative methodology [4], allowing the acquisition or improvement of skills without compromising patient safety. Simulation allows reproducing real situations to improve technical and non-technical skills, emergency decisions and critical thinking [5]. For this reason, training programs for the return of doctors into clinical practice have been including evaluation tools based on the use of simulation [2, 3, 6].

In Italy, “retraining” is based on a didactic/training system by which every healthcare professional is updated to meet the needs of patients, the organizational and operational needs of the Health Service and their own professional development. Continuous Medical Education (CME) includes the acquisition of new knowledge, skills and aptitudes for cutting-edge practice. The purpose of continuous updating is to create a system capable of promoting and verifying nationwide the quality of continuous training, including the work of independent observers and shared criteria and methods. D.Lgs 502/1992 supplemented by D.Lgs 229/1999 established the obligation of continuous training for health professionals and the National Program of CME started in 2002 [7]. Tuscany Region has drawn up guidelines for the planning of training activities by the D.G.R. 849/2002 to implement a regional accreditation system. In this system, different types of training can be accredited: residential training (RES), internship training, field training (FSC) and distance training (FAD) [8]. In the Government, Regions and Autonomous Provinces Agreement of 2 February 2017, the types of training/learning have been defined: classic residential training (RES); convention, congresses, symposia and conferences (RES); video conferencing (RES); individualized training (FSC); improvement or study groups, commissions, committees (FSC); research activities (FSC); FAD with computer/paper tools (FAD); e-learning (FAD); synchronous FAD (FAD); blended training; teaching, tutoring [9]. Although there are many types of training recognized by the ECM Program, there are not any practical teaching methods among them, so leading to training focused mainly on the teacher and less on the student [5].

Neonatal intubation is an invasive procedure, often carried out in emergency conditions, which acquires an extremely delicate meaning associated with the different degree of development of the newborn and the restricted workspace. Pediatric and newborns airways differ significantly from those of adults, especially because of larger tongue, shorter neck and jaw, smaller and more anterior airways and U-shape epiglottis (unlike the flat one in adults) [10, 11]. Simulation has proved to be an effective tool in the training of inexperienced medical personnel [12] and in retraining of experienced medical personnel on the technical skill of intubation, even in our previous research [13]. That study from our group focused on the retraining of medical personnel testing the effectiveness of a training platform consisting of a neonatal manikin sensorized in strength and position in the lower dental arch, superior dental arch and epiglottis: anatomical structures considered as the major critical point of damage during the intubation procedure [13]. Given the results and development fields of that study, in the present work we decided to sensorize the video-laryngoscope blade instead of the manikin. The blade sensorization is more accessible, as the video-laryngoscope system is an instrument already available in many clinical realities. Moreover, this choice is easier to manage and reproduce: for a matter of sensor detection efficiency, it is preferable to fix the sensors on a rigid material, such as a blade, than on soft materials such as the trachea of the manikin. In conclusion, we decided to sensorize the video-laryngoscope to provide a more accessible and reproducible instrument (Cuttano A. et al., Simulator and Sensorized System for Neonatal Intubation (S3-InNeo): planning an original bioengineering system for medical training – *submitted*).

The aim of the present study is to assess whether it is possible to increase the information and experience content of experienced medical personnel involved in the updating process, using a new sensorized instrument for the execution of the neonatal intubation procedure. The retraining platform used in this study involves the use of a software interface based on a performance evaluation game for a single skill, considering the more and more increasing use in recent years of serious games as computer/digital educational tools included in simulation [14]. Serious games are digital games with a learning purpose, therefore characterized by the union between the playful aspect and the didactic purpose [15].

## Methods

### Design and setting of study

**Fig. 1:**
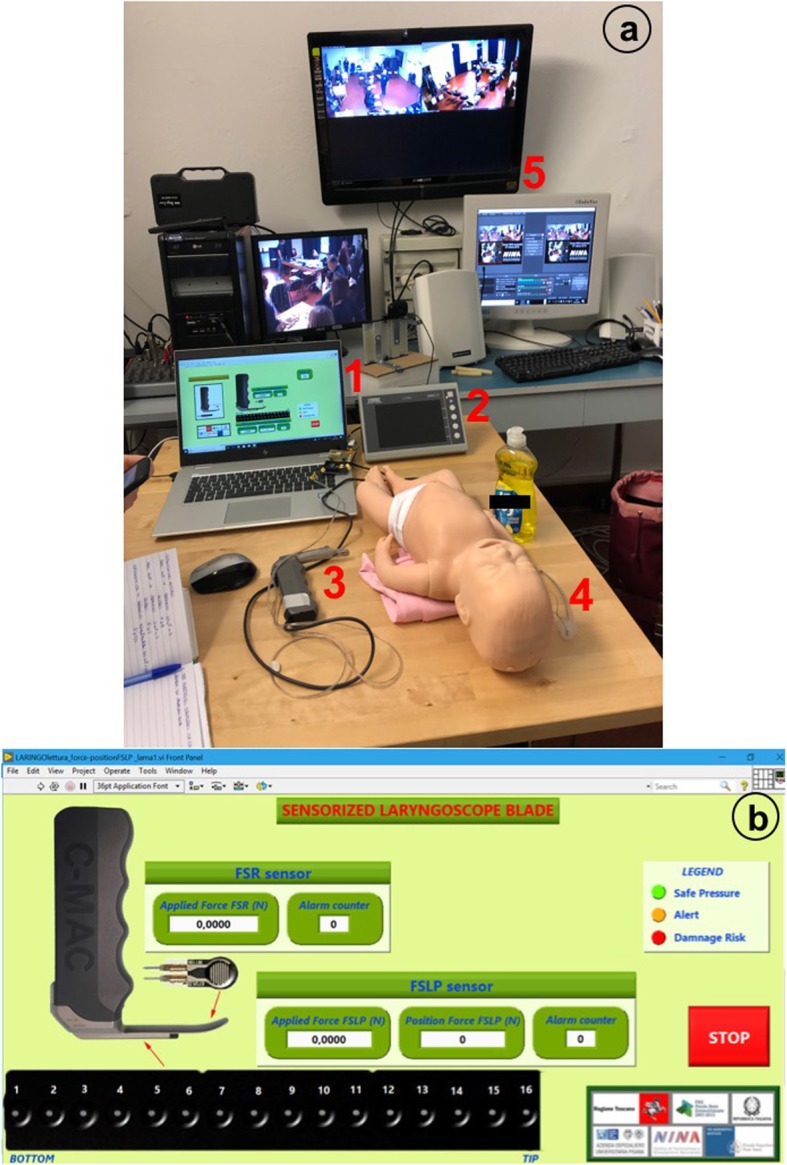
Instrumentation used in intubation test. **a** On the table you can see the user graphical interface (1), the video-laryngoscope (2) *©Storz C-Mac* with sensorized blade (3) and the manikin *©Laerdal NewBorn Anne* (4); in the background monitors (5) you can see the training course filmed by the cameras. **b** User graphical interface

We chose to use a neonatal commercial manikin©*Laerdal NewBorn Anne* and a video-laryngoscope©*Storz C-Mac* for this study. The size 1 blade of the video-laryngoscope was modified with the addition of force and position sensors that provide the user with sound and visual feedback. Such a feedback is presented through a graphical interface and gives information of applied force in the epiglottis and superior dental arch (Fig. 1).

Expert anesthesiologists and pediatricians were selected for this study: anesthesiologists have a larger experience on the intubation skill, but their experience is related to the execution of the procedure mainly on adults, so on an anatomical structure with some differences from newborn’s structures. On the other hand, pediatricians are supposed to have a better knowledge of children/neonates’ anatomical features but to have performed a total inferior number of intubation.

Anesthesiologists and pediatricians were recruited through neonatal resuscitation training courses at the Centro di Simulazione e Formazione Neonatale “NINA” (Professional Unit within the Neonatology Operational Unit of the Azienda Ospedaliero-Universitaria Pisana - AOUP) [16]: the intubation test was inserted within the course, as a form of simulation on a single skill. The groups involved in the study (see Additional file 1: Diagram S1) were homogeneous as forwards professional experience, however, the form of recruitment mediated by the training courses generated slightly different numbers of the samples themselves.

The control groups of both categories were composed of experienced medical personnel of the AOUP.

The anesthesiologists control group (Group A) was composed of five experienced doctors who performed the intubation test 4 times each, with the instrument without any kind of feedback. The threshold values set in the tests of the other groups for the anesthesiologists were extracted from this group. The following steps were carried out to obtain the thresholds, considering separately the arch and the epiglottis:

- the maximum force carried out at each intubation test was calculated for each user;
- the median of the maximum forces in all intubation tests was calculated for each user;
- the median on the results obtained among all users was extracted, obtaining the threshold value.

Experienced anesthesiologists enrolled during the training course (Group B – n° 9 people) were asked to perform the intubation test 6 times each. Unlike Group A, the instrument provided Group B members with sound and visual feedback associated with exceeding the force value set as the threshold limit. Group C was formed during another training course, composed of 10 anesthesiologists who performed the intubation test 6 times. In addition to the feedback, they were assisted by a neonatologist, expert in the neonatal intubation procedure and the use of the instrument. The purpose of this side-by-side way is to support the user to familiarize with the instrument, which has a slightly modified design compared to the original blade.

As well as for anesthesiologists, three groups were selected for pediatricians: a control group (Group A1), a group with only sound and visual feedback (Group B1) and a group with feedback plus an experienced neonatologist support (Group C1).

The only difference between the category of pediatricians and that of anesthesiologists was in the implementation of the instrumentation with a playful approach, based on the opportunity for the users to compete themselves by inserting thresholds of increasing difficulty. The “Level Game” consists of three levels of difficulty in which the set threshold varies, having been calculated from the surveys carried out on the expert control group. Indeed, Group A1 (n° 9 doctors with high experience in the neonatal intubation procedure) were asked to perform the intubation test three consecutive times, from which the thresholds to be set to the instrument were extracted. The thresholds have been defined as follows:

- the maximum force carried out at each intubation test was calculated for each user;
- the median of the maximum forces in all intubation tests was calculated for each user;
- the first quartile, the median and the third quartile were extracted from the results obtained.

The levels were defined as follows: in the first level the thresholds coincide with the value of the third quartile, so as to encourage 25% of the population to improve their performance, both in the arch and in the epiglottis; the thresholds of the second level corresponding to the value of the medians, so that 50% of the population is stimulated to further reduce their forces; the third level takes the values of the first quartile as thresholds, setting a very high target level.

The second group (Group B1), composed of 13 expert pediatricians, and the third (Group C1), consisting of 8 expert pediatricians, performed the “Level Game”, repeating the intubation test 4 times. To pass the first level, pediatricians could carry out a maximum of four over-threshold events, i.e. they can exceed the threshold twice in the arch and the same in the epiglottis; they also had to finish the procedure in less than 30 s. To move from the second to the third level they could perform only one over-threshold event in the arch and only one in the epiglottis, always intubating within 30 s. Finally, to pass the third level, they must never exceed the thresholds and always finishing the procedure in 30 s.

### Data analysis

First, the data of forces obtained from the sensors in the superior dental arch and the epiglottis were analyzed using the statistical method of Kolmogorov-Smirnov to test whether their distribution is Gaussian or not.

For each test, the forces in the epiglottis and the superior dental arch were treated separately. In both anatomical districts, the values of the forces performed by each user at each test were extracted as a median. To identify the trend of the single test, the median between the values obtained by each user in each intubation test was extracted.

After that, comparisons were made between groups (see Additional file 1: Diagram S1), comparing the medians of the forces performed by the entire group for each test:

- Comparison between Group A and Group B (Comparison A-B): made to highlight the usefulness of sound feedback in the refinement of the intubation technique, then assess whether such feedback can motivate the experienced physician to carry out ever decreasing forces;
- Comparison between Group B and Group C (Comparison B-C): carried out to assess whether the support of the doctor optimizes the confidence with the instrument, improve the performance of experienced doctors;
- Comparison between Group A1 and Group B1 (Comparison A1-B1): analogue to the comparison AB, with the only difference that Group B1 performed the procedure by receiving feedback based on the “Level Game”;
- Comparison between Group B1 and Group C1 (Comparison B1-C1): analogue to the comparison Comparison B-C, with the only difference that both groups performed the procedure by receiving feedback based on the “Level Game”;
- Comparison between Group A and Group A1 (Comparison A-A1): to assess whether anesthesiologists and pediatricians perform the intubation maneuver differently, given the considerations on the different conformation between adult and newborn.

## Results

The force data in the superior dental arch and the epiglottis obtained from the sensors in all tests had non-parametric distribution, so that Mann-Whitney test was used to assess difference between groups, with *p*-value of 0.05.

For anesthesiologists, the thresholds obtained from the control Group A were 15 N in the arch and 6 N in the epiglottis. For pediatricians, the thresholds in the superior dental arch were 20, 14 and 10 N respectively for first, second and third level; for the epiglottis were 6, 4 and 3 N.

### Comparison A-B

**Fig. 2:**
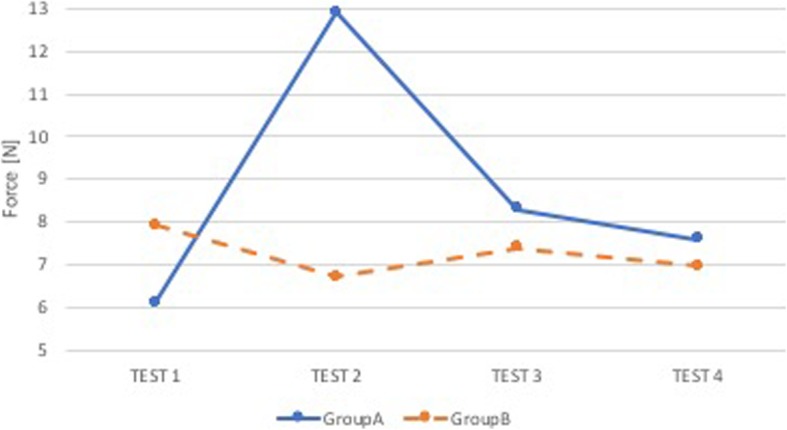
Comparison A-B: median force in the superior dental arch. Comparison between group of anesthesiologists without feedback (Group A, blue) and group of anesthesiologists with sound and visual feedback (Group B, orange). On the x-axis is the test number and on the y-axis is the median force [N] in the superior dental arch

**Fig. 3:**
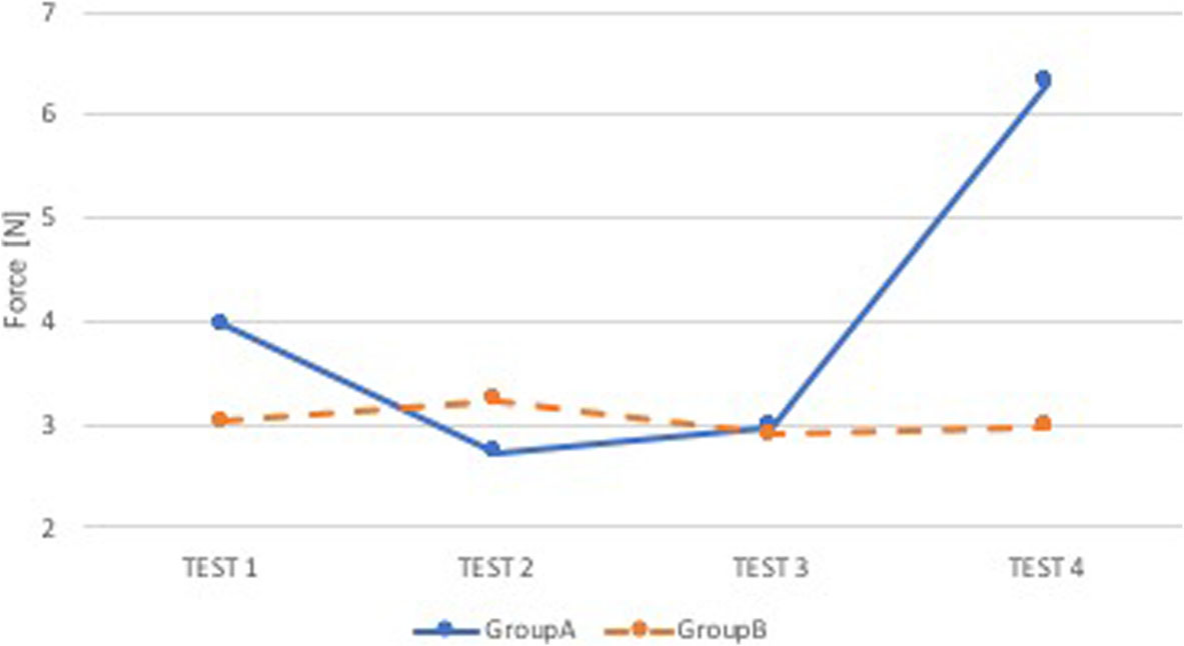
Comparison A-B: median force in the epiglottis. Comparison between group of anesthesiologists without feedback (Group A, blue) and group of anesthesiologists with sound and visual feedback (Group B, orange). On the x-axis is the test number and on the y-axis is the median force [N] in the epiglottis

Group A and Group B were not statistically different (p NS), both in terms of performance in the superior dental arch and the epiglottis, but in group B there was a slight improvement trend in the arches (Figs. 2 and 3).

### Comparison B-C

There were statistical differences between the two groups in terms of forces applied in the dental arch (*p* < 0.05), while they were not statistically different (p NS) for forces applied in the epiglottis.

**Fig. 4:**
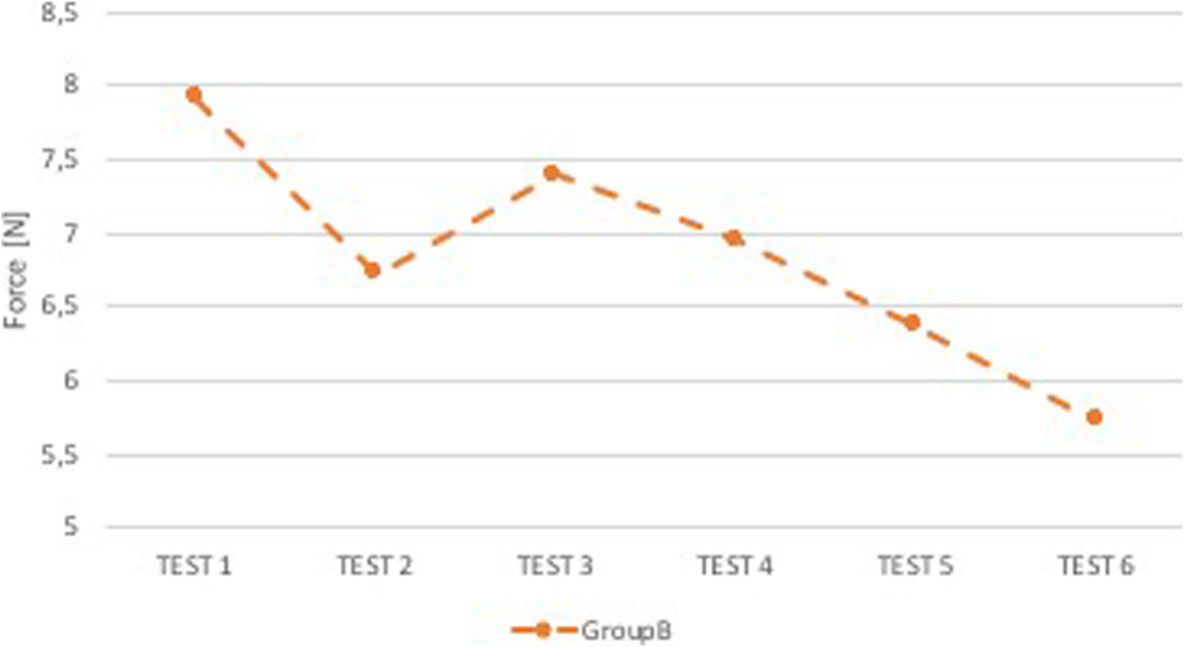
Group B: median force in the superior dental arch. On the x-axis is the test number and on the y-axis is the median force [N] in the superior dental arch. In this graph only group B, group of anesthesiologists with sound and visual feedback, is found because it is statistically different from group C, group of anesthesiologists with sound and visual feedback and support of expert neonatologist, in superior dental arch

**Fig. 5:**
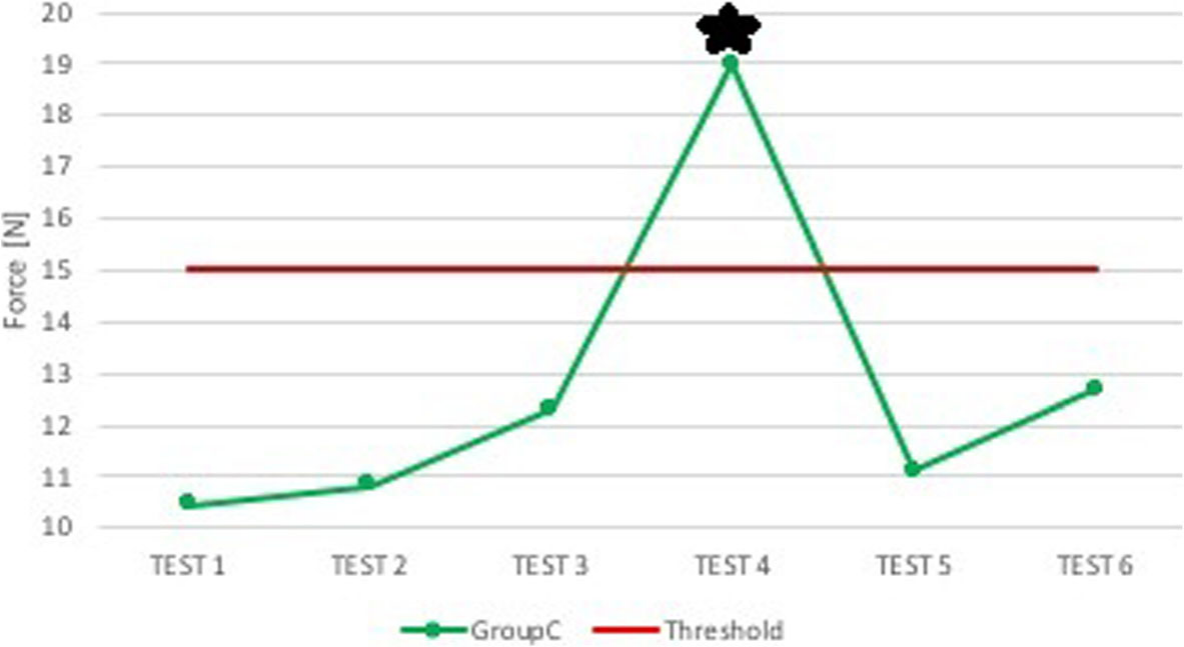
Group C: median force in the superior dental arch. On the x-axis is the test number and on the y-axis is the median force [N] in the superior dental arch (green) and the threshold value set during the tests (red). In this graph only group C, group of anesthesiologists with sound and visual feedback and support of expert neonatologist, is found because it is statistically different from group B, group of anesthesiologists with sound and visual feedback, in superior dental arch

**Fig. 6:**
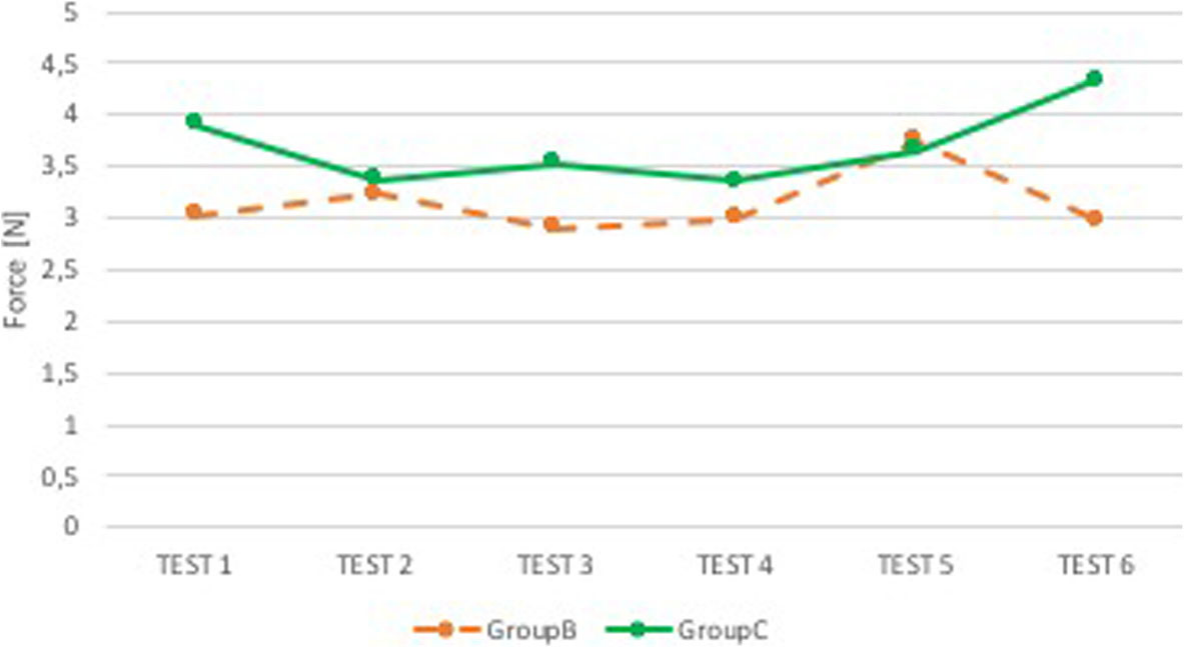
Comparison B-C: median force in the epiglottis. Comparison between group of anesthesiologists with sound and visual feedback (Group B, orange) and group of anesthesiologists with sound and visual feedback and support of expert neonatologist (Group C, green). On the x-axis is the test number and on the y-axis is the median force [N] in the epiglottis

Group B shows a trend of evident improvement in the superior dental arch (Fig. 4). Group C, on the contrary, had a tendency to higher forces along the tests, with a peak reached during the execution of the fourth one (Fig. 5). In epiglottis, for both groups, there is a fairly constant trend, with a minimal worsening associated with Group C (Fig. 6).

In both groups, the median of intubation time in the individual tests is less than 30 sec, time defined in the neonatal resuscitation algorithm [17].

### Comparison A1-B1

**Fig. 7:**
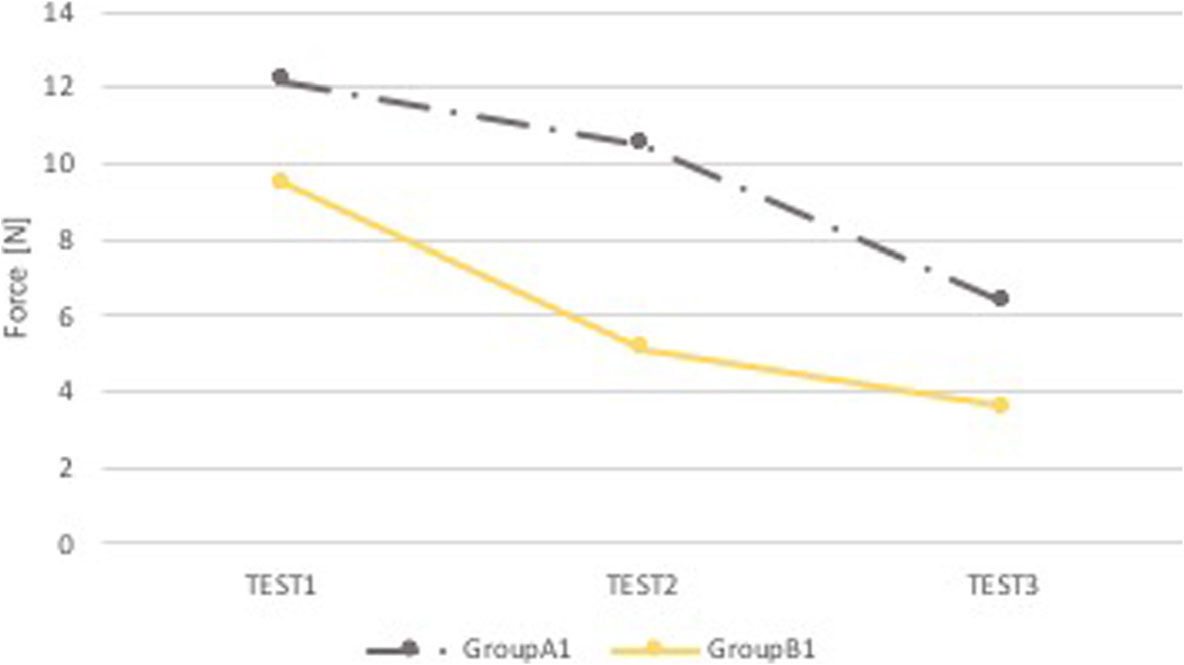
Comparison A1-B1: median force in the superior dental arch. Comparison between group of pediatricians without feedback (Group A1, grey) and group of pediatricians with sound and visual feedback (Group B1, yellow). On the x-axis is the test number and on the y-axis is the median force [N] in the superior dental arch

**Fig. 8:**
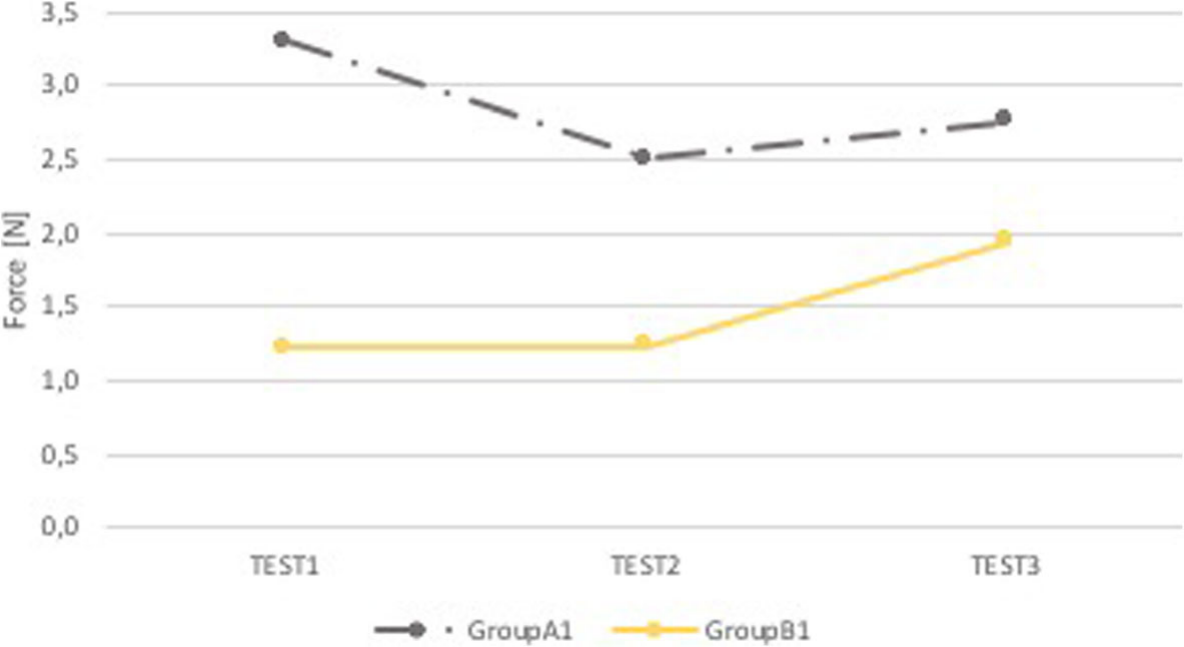
Comparison A1-B1: median force in the epiglottis. Comparison between group of pediatricians without feedback (Group A1, grey) and group of pediatricians with sound and visual feedback (Group B1, yellow). On the x-axis is the test number and on the y-axis is the median force [N] in the epiglottis

Group A1 and Group B1 have no statistically significant differences (p NS), both in the performance in the superior dental arch and the epiglottis. However, the group who received the feedback (B1) carried out slightly minor forces in both anatomical districts (Figs. 7 and 8).

### Comparison B1-C1

**Fig. 9:**
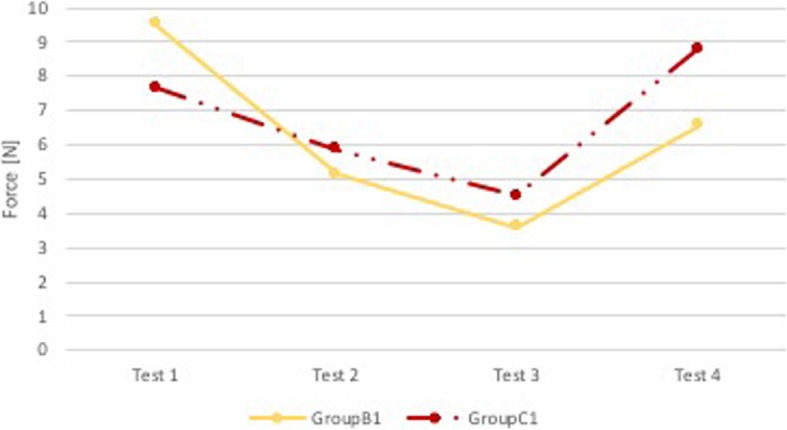
Comparison B1-C1: median force in the superior dental arch. Comparison between group of pediatricians with sound and visual feedback (Group B1, yellow) and group of pediatricians with sound and visual feedback and support of expert neonatologist (Group C1, red). On the x-axis is the test number and on the y-axis is the median force [N] in the superior dental arch

Mann-Whitney test showed that there were no statistical differences (p NS) between the two groups both in terms of performance in the superior dental arch and the epiglottis (Fig. 9). All users performed very low forces on epiglottis, below the set threshold on average; therefore the two groups did not receive feedback from the instrument (data not shown).

**Fig. 10:**
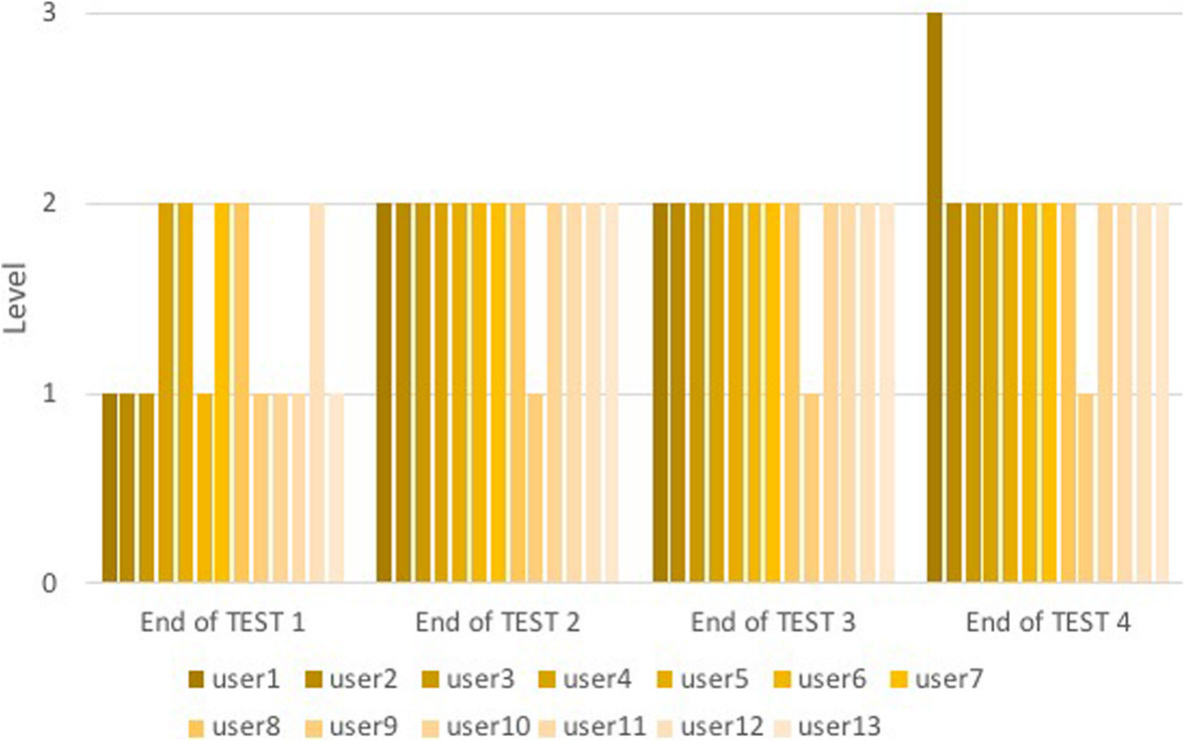
Level for each user of Group B1 at the end of each test. The columns represent the level reached by each user of Group B1 at the end of each test and are divided into four groups corresponding to the number of tests performed

**Fig. 11:**
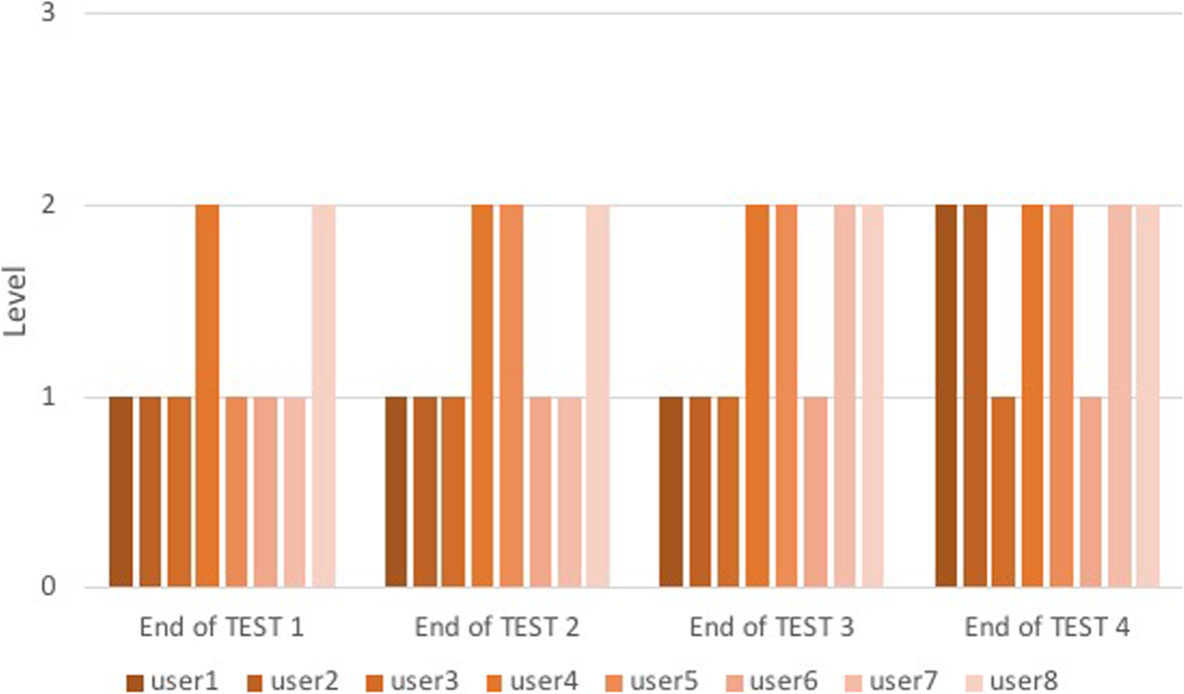
Level for each user of Group C1 at the end of each test. The columns represent the level reached by each user of Group C1 at the end of each test and are divided into four groups corresponding to the number of tests performed

The two groups ran the tests through the “Level Game”, but not all users passed the level at the same test, so the number of over-threshold events was not compared. Except for one, all users in Group B1 passed the first level to the second intubation (Fig. 10); only one passed the second level to the last intubation. Almost the entire Group C1, except for two users, passed the second level in the four-intubation tests performed (Fig. 11).

The median of intubation time in individual tests was less than 30 sec in both groups.

### Comparison A-A1

**Fig. 12:**
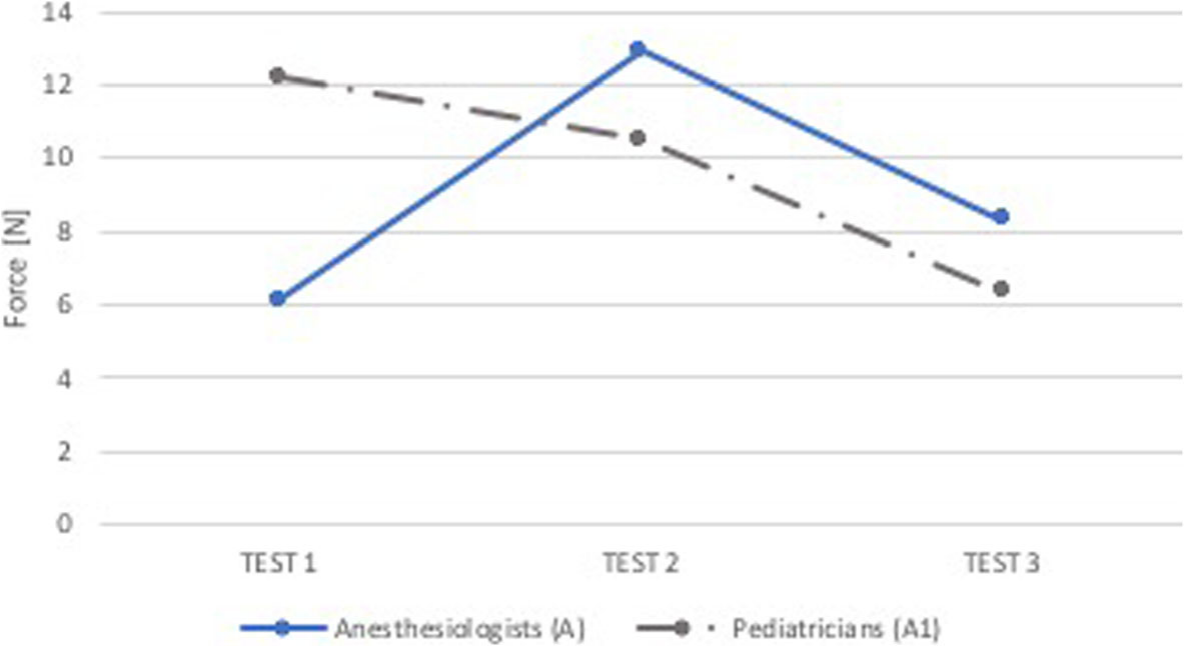
Comparison A-A1: median force in the superior dental arch. Comparison between group of anesthesiologists without feedback (Group A, blue) and group of pediatricians without feedback (Group A1, grey). On the x-axis is the test number and on the y-axis is the median force [N] in the superior dental arch

**Fig. 13:**
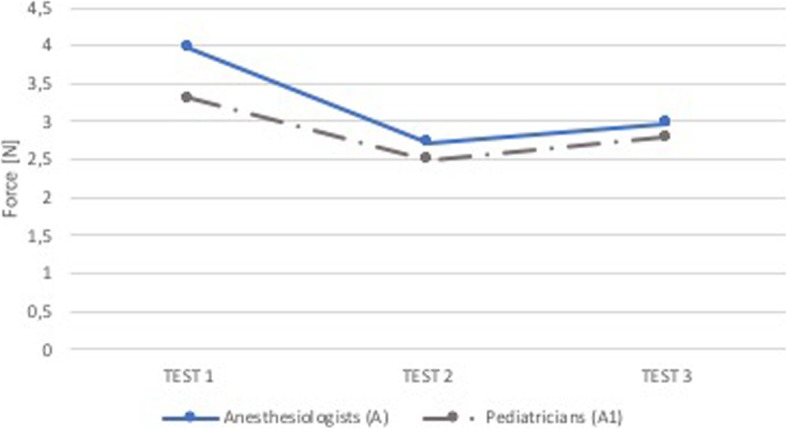
Comparison A-A1: median force in the epiglottis. Comparison between group of anesthesiologists without feedback (Group A, blue) and group of pediatricians without feedback (Group A1, grey). On the x-axis is the test number and on the y-axis is the median force [N] in the epiglottis

As shown in Figs. 12 and 13, except for forces applied in the superior dental arch in the first test, the two categories of experienced physicians carried out forces very similar to each other (no statistically significant differences - p NS).

## Discussions

The results showed that control groups did not present a specific trend, but rather a random trend, probably due to the lack of any kind of feedback on the forces applied.

Analysis of anesthesiologists’ data showed a slight improvement in performance carried out with sound/visual feedback only: we speculate that this may be due to the fact that an expert on intubation maneuver, like the anesthesiologist, didn’t receive any benefit in the use of an instrument not able to trig a real challenge to the refinement of the procedure, even if sensorized. Moreover, as forward the result of the not-improving trend of the group with the support of the neonatologist, it could be caused by the fact that the physician, who accompanied users during the procedure, influenced the intubation style, trying to vary in a few attempts a technique that the anesthesiologist has perfected and consolidated throughout his own experience along years.

Among pediatricians, on the contrary, feedback seems to benefit. Pediatricians involved in this study, although having many years of experience in clinical practice, represent a less experienced category on intubation maneuvers than the anesthesiologist, considering only the number of annually conducted intubation procedures. The presence of the Tutor does not seem to have greatly affected the performance of pediatricians, who performed slightly higher forces in the dental arch than the group only with sound/visual feedback, performing many over-threshold events. On epiglottis, the median forces were much lower than the thresholds: indeed, the over-threshold events were detected very rarely. The result obtained by expert pediatricians is likely to be associated with the manikin conformation which induces the physician to perform a rotational movement of the wrist, for design reasons, while in vivo this procedure is performed with the maintenance of the wrist fixed and bringing forward the blade to lift the epiglottis. The manikin is also composed of materials more rigid than in vivo tissues.

Pediatricians appreciated the “Level Game”, as a challenge trying to overcome their previous performances. This appreciation was confirmed by the results obtained from the questionnaire they completed at the end of the test (data not shown). As reported (Figs. 10 and 11) most of the users reached the second level, a high objective considering especially the few over-threshold events that could be done to move to the next level. This additional playful approach was based on the rationale that Pediatricians are supposed to be less confident than Anesthesiologists with intubations skills. Of course, on one hand the “Level Game” challenge was a tool to collect more important data for us; on the other hand we could consider the slight difference among Pediatricians and Anesthesiologists as a methodological limitation of the study. However, in our opinion, advantages in terms of deeper observation outweigh the disadvantages.

In all groups, it is often noted that performance decreases after the fourth intubation, probably due to both physical and mental fatigue.

## Conclusions

We can conclude that in our study the sensorized neonatal intubation instrument, which emits sound feedback was more effective for pediatricians than for anesthesiologists. We can speculate that pediatricians performed lower forces, especially on the epiglottis, because they were used to treat children, who have tissues with sensitivity and conformation different to adults; while anesthesiologists tended to complete the procedure as soon as possible, even if performing higher forces.

In addition, we can say that the inclusion of the “Level Game” is more effective than the use with only feedback, as a form of training for increasing levels where an experienced user can compete with himself.

The support of an expert Neonatologist to facilitate the knowledge of the instrument during the execution is more effective with less experienced personnel. Indeed, for an expert physician, it is more efficient to adapt the instrument to their technique than to adapt their technique to the instrument, because they have an intubation methodology trained and consolidated during their own experience.

In sum, the system is effective for the retraining of experienced medical personnel, if made playful and competitive and if the users can adapt their technique to the instrument in total autonomy, respecting their own technique and timing, without any outside help.

A future idea could be to adapt this instrument for intubation for clinical activity, as a tool to help maintaining low forces carried out on the most stressed neonatal anatomical districts during the intubation procedure.

## Supplementary information

**Additional file 1:** Diagram S1. Summary of anesthesia and pediatric groups and comparisons between them.

## Data Availability

All data generated or analysed during this study are included in this published article (and its supplementary information files).

## Abbreviations

AOUP: Azienda Ospedaliero-Universitaria Pisana
CME: Continuing Medical Education
FAD: Distance training
FSC: Field training
RES: Residential training
