# Supplementary material for: Effectiveness of a new sensorized videolaryngoscope for retraining on neonatal intubation in simulation environment
Source: Ital J Pediatr. 2020 Feb 3;46:13. doi: 10.1186/s13052-020-0774-z (PMC6998091; doi:10.1186/s13052-020-0774-z)
Supplement: Supplementary file 1 — Additional file 1: Diagram S1. Summary of anesthesia and pediatric groups and comparisons between them. [file 13052_2020_774_MOESM1_ESM.pdf]

ANESTHESIOLOGISTS

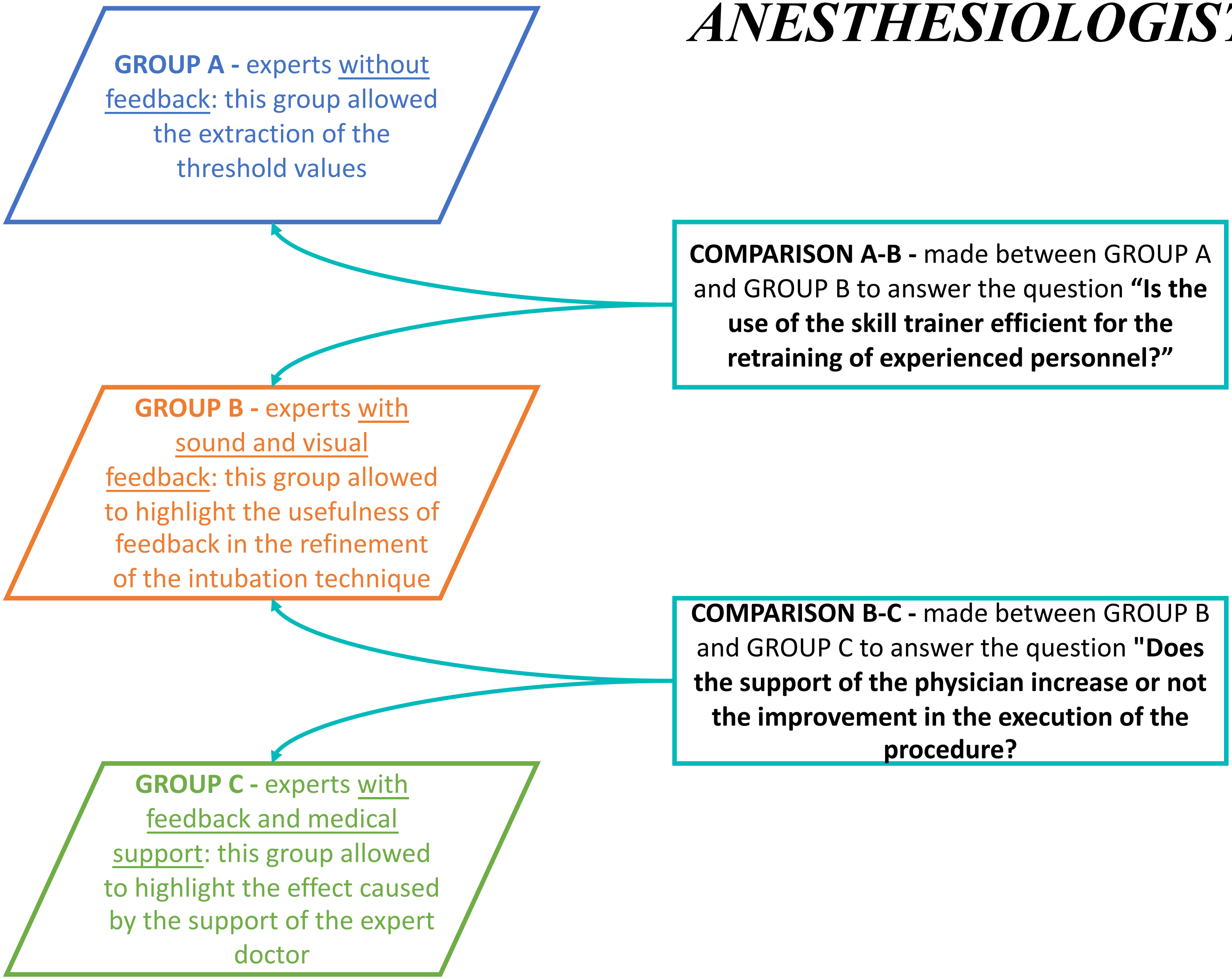

PEDIATRICIANS

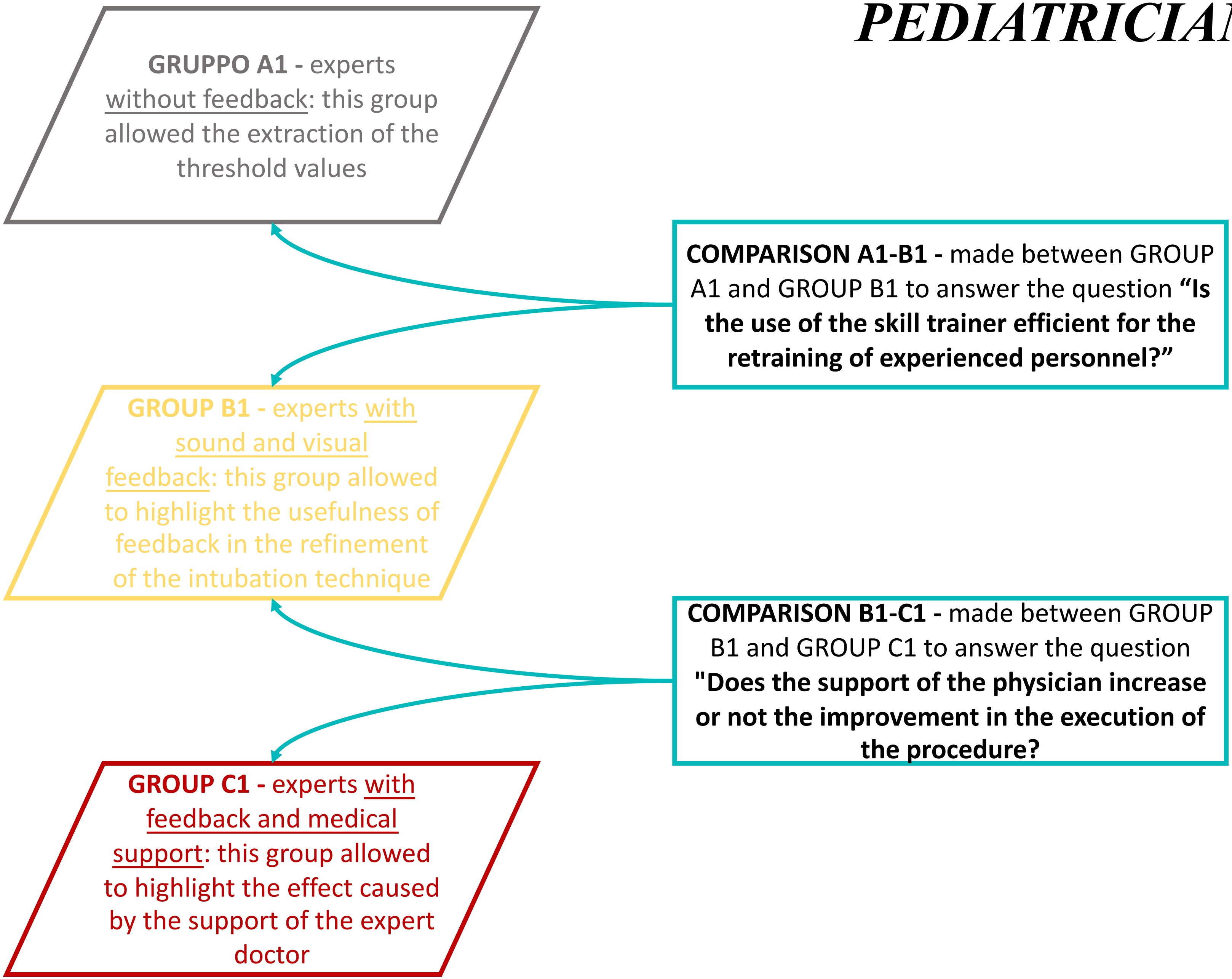

ANESTHESIOLOGISTS vs PEDIATRICIANS

GROUP A – <u>anesthesiologists</u> without feedback: this group allowed the extraction of the threshold values

COMPARISON A-A1 - made between GROUP A and GROUP A1 to assess whether anesthesiologists and pediatricians perform the intubation maneuver in a different way

GRUPPO A1 - <u>pediatricians</u> without feedback: this group allowed the extraction of the threshold values
